# A New Localization System for Indoor Service Robots in Low Luminance and Slippery Indoor Environment Using Afocal Optical Flow Sensor Based Sensor Fusion

**DOI:** 10.3390/s18010171

**Published:** 2018-01-10

**Authors:** Dong-Hoon Yi, Tae-Jae Lee, Dong-Il “Dan” Cho

**Affiliations:** 1 Department of Electrical and Computer Engineering, Automation and Systems Research Institute (ASRI), Seoul National University, Seoul 151-742, Korea; ydh01@snu.ac.kr (D.-H.Y.); ltj88@snu.ac.kr (T.-J.L.); 2 Inter-University Semiconductor Research Center (ISRC), Seoul National University, Seoul 151-742, Korea

**Keywords:** AOFS, localization, low illumination, slippery environment

## Abstract

In this paper, a new localization system utilizing afocal optical flow sensor (AOFS) based sensor fusion for indoor service robots in low luminance and slippery environment is proposed, where conventional localization systems do not perform well. To accurately estimate the moving distance of a robot in a slippery environment, the robot was equipped with an AOFS along with two conventional wheel encoders. To estimate the orientation of the robot, we adopted a forward-viewing mono-camera and a gyroscope. In a very low luminance environment, it is hard to conduct conventional feature extraction and matching for localization. Instead, the interior space structure from an image and robot orientation was assessed. To enhance the appearance of image boundary, rolling guidance filter was applied after the histogram equalization. The proposed system was developed to be operable on a low-cost processor and implemented on a consumer robot. Experiments were conducted in low illumination condition of 0.1 lx and carpeted environment. The robot moved for 20 times in a 1.5 × 2.0 m square trajectory. When only wheel encoders and a gyroscope were used for robot localization, the maximum position error was 10.3 m and the maximum orientation error was 15.4°. Using the proposed system, the maximum position error and orientation error were found as 0.8 m and within 1.0°, respectively.

## 1. Introduction

Indoor service robots co-exist with people in offices, schools, and homes, and provide specific services. In an indoor space, a service robot is required to accurately estimate its position to fulfill its mission efficiently. A wheel-based indoor mobile robot basically estimates its position by integrating sensors such as wheel encoders or inertial sensors. However, its distance and orientation error accumulate as the robot moves.

To solve this problem, several studies have been performed with the help of external equipments, including position estimation from the attenuated signal intensity according to the distance from wireless access point [1,2], triangulation of location by installing multiple ultra-wideband beacons [3] or using together with building indoor structure information [4], image processing by installing multiple cameras in an indoor space [5], and printing bar codes on floor or attaching radio frequency identification tags [6,7]. However, these methods are inconvenient, since equipments must be installed and calibrated in advance. Therefore, consumer service robots mainly use built-in sensors such as wheel encoders, inertial sensors, mono camera, stereo camera, RGB-D camera [8,9] or laser scanner [10,11] for localization without external assistance. Among vision sensors and laser scanner, mono camera is highly attractive owing to its low cost, light weight, and low power consumption.

**Figure 1:**
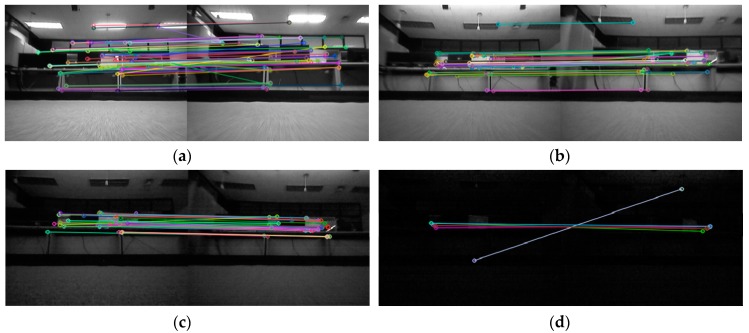
Oriented fast and rotated brief feature matching results under specific illumination conditions. (**a**) 120.0 lx; (**b**) 12.0 lx; (**c**) 2.0 lx; (**d**) 0.3 lx.

Recently, simultaneous localization and mapping (SLAM) techniques for localization using a mono camera have been proposed [12,13]. However, in low illumination condition, the amount of light reaching image sensors decreases. The feature extraction and matching performance deteriorate, as shown in Figure 1, and conventional vision based localization methods are difficult to be operated. Furthermore, when conventional wheel encoders are used, localization error is significantly increased when a robot slides in a slippery environment [14,15]. Typically, slippage which occurs on carpets, rugs, and thresholds is hard to be recognized by inertial sensors reliably.

In this paper, we propose a system for robot localization in a low illumination and slippery environment by combining low-cost motion sensors, an afocal optical flow sensor (AOFS), and a forward-viewing camera. Unlike previous work, AOFS for robot localization is adopted in a slippery environment. Also, the interior space structure from an image and robot orientation is estimated. In this work, the target illumination condition was approximately 0.1 lx, where conventional vision-based SLAM method is not at all efficient. Instead of the conventional point feature extraction and matching for localization, the interior structure of environment from an image for robot orientation estimation is assessed. To achieve this, a rolling guidance filter is applied to eliminate image noise and enhance the appearance of image boundary after applying histogram equalization to brighten the image. Then, the vanishing point is extracted to figure out the dominant orientation of interior space.

The organization of this paper is as follows. The related works are briefly reviewed in Section 2. The primary methods are then introduced in Section 3. Section 4 presents the experimental process and Section 5 discusses and analyzes the experimental results. Finally, the study is concluded in Section 6.

## 2. Related Works

### 2.1. Slippage Detection Methods

The most typical algorithm for slip detection is to estimate the displacement of a robot by processing output information of wheel encoders and inertial sensors with the Kalman filter. However, since this method has an error resulting from the double integration of an accelerometer, position error increases as the moving distance increases [16]. Also, an accelerometer needs to be adjusted to zero in the stop state. After adjustment, error varies depending on the ambient temperature or gravity [17].

To solve this problem, an optical mouse, which has been popularized since late 1990s, attracted great attention in robot localization [18,19,20,21]. The principle of an optical flow sensor (OFS) mounted on an optical mouse is that, when illumination generated from a light emitting diode is irradiated on floor surface, light reflected on the surface is condensed by a lens and collected by an image sensor. At this time, a digital signal processor in OFS analyzes the flow of consecutively collected images to calculate its x- and y-axis movement displacements [22].

In the early 2000s, a variety of experiments were performed with OFS to determine the operating characteristics varying floor material, OFS height from floor, light amount irradiated on the bottom, and moving speed of OFS, among others. The part which needs to be improved for use as a robot position estimation sensor was clarified by the height with respect to the floor where OFS operation is guaranteed [20,23]. Since the height guaranteed for OFS operation is within 2.7 mm, it is necessary to mount OFS below a robot as close as possible to the floor, which limits the height of obstacles a robot can get over while moving. At the same time, reliable data cannot be obtained when slippage occurs on carpets and thresholds, among others, where height varies to a great extent. Various attempts have been made to overcome this issue, which includes minimizing position measurement dispersion using 2–8 OFS [21,24,25] and mounting multiple OFSs with an increased focal length [26,27]. The conversion factor from OFS’s moving counts to travel distance, however, also varied as distance change from floor to OFS. Therefore, it was difficult to estimate the moving distance accurately. Hyun et al. propose the arrangement of two OFSs different heights as a method of obtaining the moving distance regardless of the variation in height [28]. However, since this method requires two OFSs, there is an image blurring phenomenon because of the change in height between floor and sensor. In addition, the fabrication of optical isolator is also difficult and expensive.

In our previous work [29], AOFS system for odometry estimation was proposed. To reduce error from vertical height variance, infinite effective focal length system was applied. An afocal system coincides with the front focal point of the second system; this makes the rays parallel to the horizontal axis in object space conjugate to the rays parallel to the axis in image space. The AOFS can robustly estimate the moving distance of a robot with a height changeover of 2.7 mm. In this work, we adopt AOFS for moving distance estimation of a robot on carpets which induces wheel slippery.

### 2.2. Image Enhancement under Low Luminance

**Figure 2:**
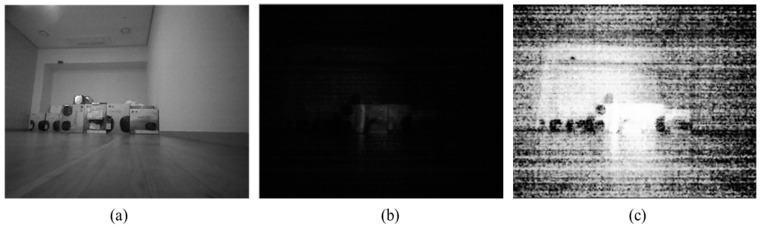
Histogram equalization result of a low-light image around 0.1 lx. (**a**) Image taken with an indoor light turned on; (**b**) Indoor light turned off; (**c**) Histogram equalization of image in Figure 2b.

Several studies have been conducted to improve the brightness of low-illuminance images [30,31,32,33,34,35]. Histogram equalization, contrast limited adaptive histogram equalization [33] and retinex algorithm [34,35] are the most useful methods. To improve the global contrast of an image, the most frequent intensity values should be spread out effectively. However, when this method is applied to a dark image, there is an adverse effect which generates severe noise, as shown in Figure 2. This paper uses histogram equalization to enhance the image brightness.

### 2.3. Identifying Indoor Structure Using a Single Image

The use of vanishing points helps to grasp spatial information [36,37,38,39,40]. In general, parallel straight lines in an image often exist in an artificial structure. If straight lines in the structure are found and the points where they converge are found as vanishing ones, the interior spatial structure can be grasped with one image. The assumption that line segments detected in an indoor space are mainly perpendicular or parallel to each other is called the Manhattan frame assumption. Using this assumption, we can extract three vanishing points (VPs) perpendicular to each other in the image.

A vanishing point is an abstract one on an image plane where parallel straight lines converge in a process of projecting a three-dimensional space into a two-dimensional image. In the case of a rectangular parallelepiped, lines which compose it can be forcibly aligned in three directions. In addition, if a line is expanded into an infinite space, it is gathered at the points formed in a 2D plane. Each vanishing point on a 2D plane can be represented by a normal vector. In addition, since each vanishing point is perpendicular to each other, if two vanishing points can be obtained, the remaining vanishing point can also be obtained.

## 3. Proposed Methods

In this paper, we propose a method which can estimate the position of an indoor service robot in a low luminance and slippery environment of approximately 0.1 lx, where conventional vision-based SLAM methods are hard to be operated. Wheel encoders, a gyroscope, AOFS, and a mono camera are used for robot localization.

If an image from a mono camera is too dark, it is difficult to operate SLAM because the feature extraction and matching quality are degraded, as shown in Figure 1d. If histogram equalization is applied to improve the image contrast, the image noise increases, which makes the image difficult to use. At this time, if rolling guidance filtering [41] is applied, although lines are not abundant, it can be used for azimuth estimation. Straight line segments extracted from an image are classified into similar directions. Three directions orthogonal to each other are obtained to estimate the vanishing point. Using the extracted vanishing point, a method to estimate the difference in angle between space and robot is proposed and is used to correct robot’s azimuth angle.

**Figure 3:**
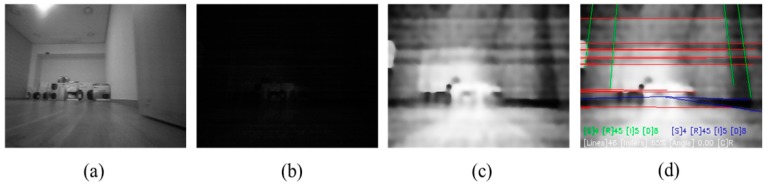
Low-light image enhancement and vanishing point extraction process. (**a**) Indoor image taken with light turned on; (**b**) Image taken with light turned off; (**c**) Rolling guidance filtering after histogram equalization applied to the low-luminance image of Figure 3b; (**d**) Spatial angle acquisition by extracting line and estimating vanishing point of Figure 3c.

Figure 3a shows the indoor image taken with light turned on, while Figure 3b shows the image when illumination is turned off. Figure 3c shows the image when rolling guidance filtering is applied after histogram equalization to the low luminance image in Figure 3b. Figure 3d shows the result of extracted VPs using the image in Figure 3c.

**Figure 4:**
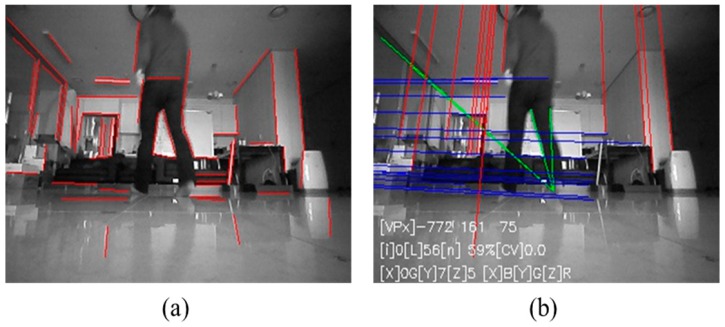
Line segments in various directions. (**a**) Line segments extraction image; (**b**) Image of straight lines extracted that constitute the vanishing point.

A general indoor image is not composed of only straight lines perpendicular to each other, as shown in Figure 3c. However, it is composed of straight lines in various directions, as shown in Figure 4. Therefore, to extract the straight line constituting VPs, a direction histogram is calculated for each straight line extracted from the image. A set of straight lines corresponding to the direction common to line plurality is called set “A”. A set of lines not belonging to set “A” is called set “B”. It is assumed that the two sets are orthogonal to each other. Then, lines satisfying orthogonal assumptions are selected by a random sample consensus, and the vanishing point is estimated. At this time, the vanishing point of the straight line belongs to the plane having the straight line direction as a normal vector. The result of the scalar product of plane normal vector and line vector is smaller than the threshold value. Subsequently, using the Levenberg–Marquardt algorithm, the vanishing point vector which minimizes the energy is finally selected [37]. Figure 4a shows the straight lines extracted from the image, while Figure 4b leaves only the straight lines which make up the vanishing point.

**Figure 5:**
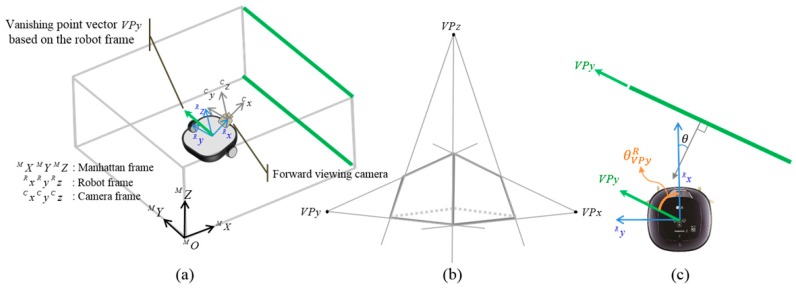
Frame relation and vanishing points. (**a**) Manhattan, robot, camera frame definition; (**b**) Three vanishing points from a perspective scene; (**c**) Theta is the angle between *^Y^M* and *^R^X*.

**Figure 6:**
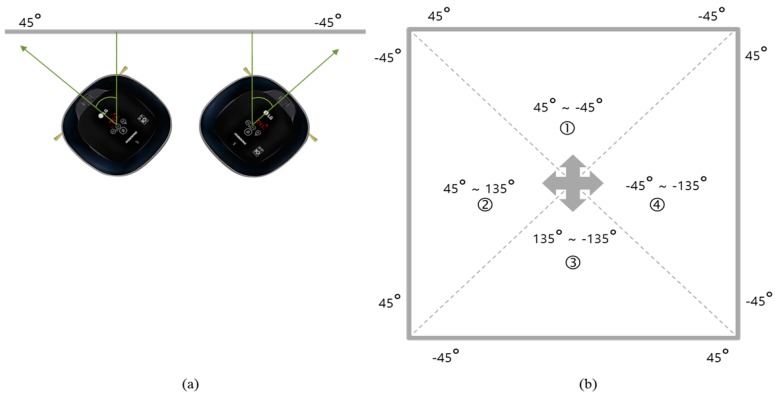
Manhattan frame based and robot based azimuth conversion relationship. (**a**) Manhattan frame based azimuth; (**b**) Robot frame based azimuth.

When the indoor structure is grasped by estimating the vanishing point in the image with an improved contrast and sharpness, the robot orientation (robot azimuth angle) with respect to the indoor space (also called as a Manhattan frame) can be obtained. From the first image taken from the camera on the robot, robot orientation with respect to indoor space can be estimated. In the case where orientation error from the gyroscope increases while the robot is moving, it can be used for azimuth correction of the robot using the relative angle with respect to the indoor space. In this work, the forward-viewing camera is slightly tilted by 8.7° to acquire more information. Using the simple fixed rotation matrix, the direction vector of vanishing point with respect to camera frame is converted to the robot referenced coordinate as follows:

(1) [VRP1xVRP2xVRP3xVRP1yVRP2yVRP3yVRP1zVRP2zVRP3z]=[1000cos(tilt)sin(tilt)0−sin(tilt)cos(tilt)][VcP1x′VcP2x′VcP3x′VcP1y′VcP2y′VcP3y′VcP1z′VcP2z′VcP3z′]

where *^R^VP* and *^c^VP* are direction vectors of vanishing point with respect to the robot frame and the camera frame, respectively. As the robot rotates only in the Z-axis direction with respect to the robot frame, a vanishing point having the largest X-axis direction coefficient is selected and an angular difference from direction vector of the robot is calculated. The directions of x, y, and z are illustrated in Figure 5a. Three VPs from a perspective scene are shown in Figure 5b. The relation between *VP_x_* and ^R^X is shown in Figure 5c. The range of azimuth angle estimated from the vanishing point is from −45° to 45°, whereas the robot orientation is from −179° to 180°. As shown in Figure 6b, the range of current robot azimuth (①, ②, ③, and ④) is checked. When robot azimuth exists in the interval of *n* (*n* is 1–4), by adding (*n* − 1) × 90° to VP estimation azimuth, the robot azimuth angle with respect to world frame can be calculated. The estimated robot azimuth angle is adopted only when the difference between robot azimuth angle before correction and the azimuth corrected by vanishing point is within 20°, thereby ensuring stability in azimuth correction.

**Figure 7:**
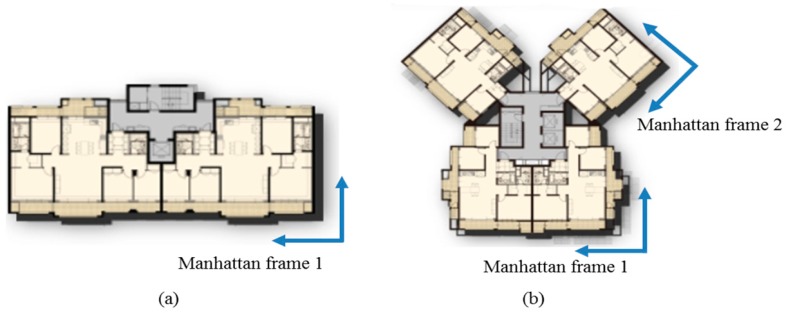
Example of Manhattan frame configuration in a blueprint of typical home environment. (**a**) Indoor environment which can be modeled using a single Manhattan grid; (**b**) Indoor environment which can be modeled using multiple Manhattan grids.

The above description deals with the case where the indoor environment consists of a single Manhattan frame, as shown in Figure 7a. However, it is difficult to deal with multiple Manhattan spaces, as shown in Figure 7b. To cope with these multi spaces, we assume that the indoor space can have multiple reference directions. Using the direction vector of vanishing point extracted from horizontal lines, $\theta_{VPy}^{R}$ can be calculated, which is the relative angle between the robot and horizontal line segment in front of it. The direction of vanishing point with respect to world frame can be calculated as shown in Equation (2).

(2) $$\theta_{VPy}^{W}=\theta_{VPy}^{R}+\theta_{robot}^{W}$$

where $\theta_{robot}^{W}$ is the robot angle with respect to the world frame. Once a number of world based vanishing point angle samples are obtained, average angle ${\bar{\theta }}_{VPy}^{W}$ is stored in the database. At this time, the direction of indoor space is registered only when the difference is larger than a predetermined angle with previously stored vanishing point direction. Using the current observation of $\theta_{VPy}^{W}$, if the condition of $\left|\theta_{Vpy}^{W}-{\bar{\theta }}_{VPy}^{W}\right|<\;threshold$ is satisfied, it is regarded that the vanishing point direction in the database is detected in the current image frame. In this case, the robot angle with respect to the world frame $\theta_{robot}^{W}$ can be calculated as shown in Equation (3), and the spatial relationship between robot and space is shown in Figure 5c.

(3) $$\theta_{robot}^{W}={\bar{\theta }}_{VPy}^{W}-\theta_{VPy}^{W}$$

Normally, when a slippage occurs by the robot’s own force, the amount of change in the wheel encoder becomes larger than the one measured by AOFS. However, since external force acts on a robot during moving, the robot may be pushed without turning wheels. Especially, when a robot runs on a carpet, side slip occurs due to elastic effect of the carpet. Therefore, when a difference of more than a certain level occurs in the position variation amount measured by wheel encoder and AOFS, it is determined that slip has occurred. If slip does not occur, the wheel encoder output data is more accurate than AOFS. If slip occurs, the moving distance is estimated by weighting the data from AOFS. The proposed robot localization algorithm is shown in Algorithm 1. At every 10 ms, robot moving displacement fused from AOFS and wheel encoder is combined with angular information acquired from the gyroscope to generate continuous relative position information of $\Delta X_{odo}={\left(\Delta x_{r},\Delta y_{r},\Delta \theta_{gyro}\right)}^{T}$. However, the angle information estimated by using vanishing points from improved dark images is provided in a discontinuous manner. At this time, robot trajectories are optimized so as to minimize the objective function, as shown in Equation (4). The Lebenberg–Marquart algorithm is used for optimization.

(4) $$E(x_{c,s}^{w},\cdots ,x_{c,k}^{w})=\sum_{i}\|(\Delta X_{odo,i}-(x_{i}^{w}⊖x_{i-1}^{w})){\|}^{2}+\sum_{i}{\left(\theta_{robot,i}^{W}-\theta_{i}\right)}^{2}$$

where $x_{i}^{w}$ is the position of the *i*-th robot, which is displacement, angle $\Delta X_{odo,i}$ is the odometry measurement, which is the relative pose between the (*i* − 1)-th camera pose and *i*-th camera pose, ⊖ is the inverse pose composition operator, $\theta_{robot,i}^{W}$ is the *i*-th VP-based robot orientation measurements, and $\theta_{i}$ is the *i*-th camera orientation estimation.

**Algorithm 1.** Robot localization algorithmInput:AOFS, wheel encoders, a gyroscope, and a foward viewing imageOutput:Optimized robot pose trajectory1:**For** every samples **do**2: generate odometry data with the AOFS, wheel encoders, and the gyroscope3: apply rolling guidance filter after histogram equalization to the image4: extract vanishing points from the image5: estimate robot azimuth from the vanishing points 6: optimization robot pose trajectory using the Lebenberg–Marquard algorithm 7: return the optimized robot pose trajectory8:**end For**

## 4. Experiments

**Figure 8:**
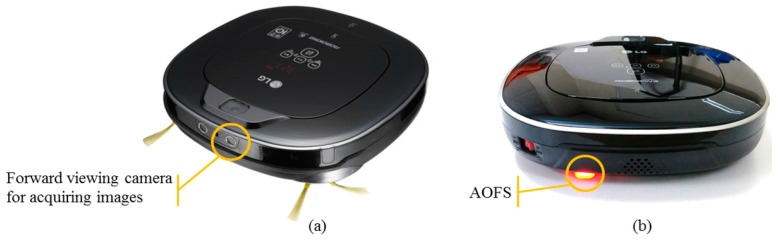
Robot platform for experiments. (**a**) Forward viewing camera is located in the front of the robot; (**b**) AOFS is located on the bottom left of the robot rear.

During the practical application of robot, the image of forward-viewing camera and the odometry sensor data are stored in the robot memory, acquiring the test image data set, after that the preliminary verification is performed through computer simulation. Subsequently, position estimation experiment is performed by porting the proposed algorithm to ARM Cortex A9 board, which is connected to the robot. A LG Hom-Bot VR6480VMNC (LG Electronics, Seoul, South Korea) equipped with a forward-viewing camera, wheel encoders, AOFS, and a gyroscope is used, as shown in Figure 8.

**Figure 9:**
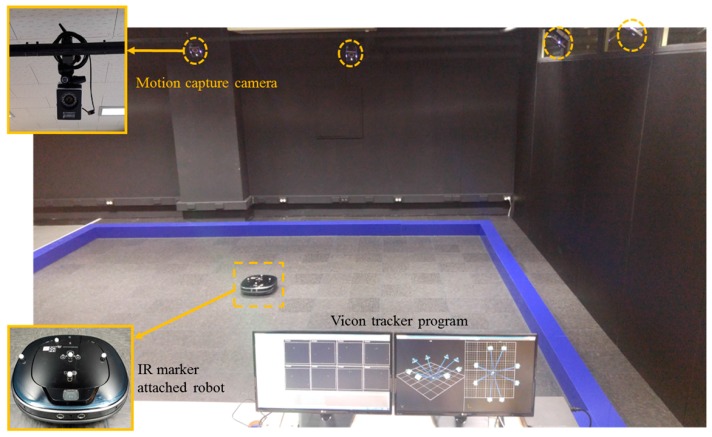
The experimental environment for robot tracking with Vicon motion captures system.

The Vicon motion capture system (Vantage V5, VICON, England) is used to measure the true position of the robot in an indoor space where the illumination can be controlled. The motion capture system tracks the position of infrared reflective markers with high accuracy, that is, with less than 0.5 mm error. The Vicon motion capture system is illustrated in Figure 9.

**Figure 10:**
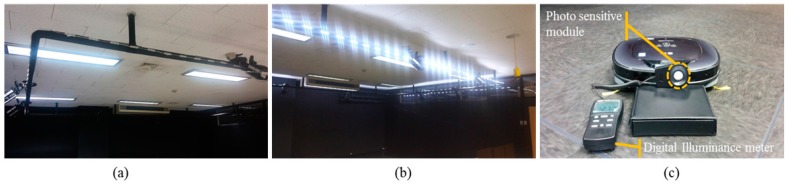
Illumination control system consisting of LED bars which attached outward of the ceiling square frame. (**a**) LED turned off; (**b**) LED turned on; (**c**) Illuminance is measured in front of the forward viewing camera using the digital illuminance meter.

Initially, to check the low illumination condition, a LED illumination system which can adjust the brightness is setup, as shown in Figure 10. The result of the feature point extraction and matching performance under various illuminations are tested. The illuminance is measured by a digital illuminance meter (TES-1335, TES, Taipei, Taiwan) capable of light measuring levels ranging from 0.01 to 100 lx with an accuracy of 3%, and the test results are shown in Figure 1.

**Figure 11:**
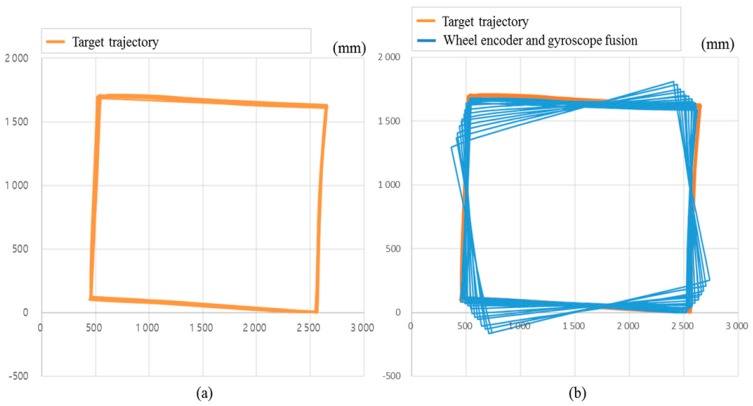
Estimated robot trajectory with wheel encoders and a gyroscope in nonslip environment captured by Vicon motion capture system. (**a**) Target trajectory; (**b**) Blue line represents the robot trajectory while driving 20 times in a counterclockwise direction along a 1.5 m × 2.0 m rectangular area.

**Figure 12:**
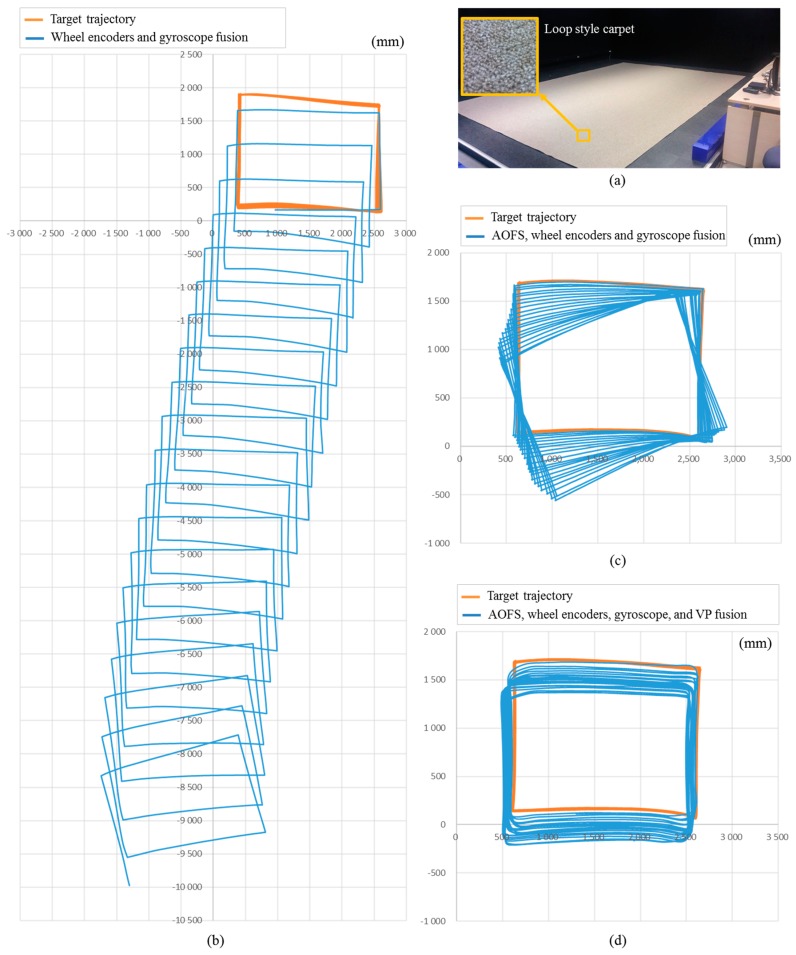
Comparison of robot trajectory estimation performance in carpeted low-light environment at 0.1 lx. (**a**) Indoor experimental environment partially covered with loop-style carpet; (**b**) Estimated robot trajectory by wheel encoders and a gyroscope which is a conventional method; (**c**) Robot trajectory by AOFS, wheel encoders, and a gyroscope not using VP; (**d**) Experimental results using the proposed method.

In a low-illuminance environment of 0.1 lx, Figure 11 shows the result of position estimation of the robot using a wheel encoder and a gyroscope while driving the robot 20 times in a counterclockwise direction along a 1.5 × 2.0 m rectangular area without a carpet. The average position error and standard deviations are 175 and 114 mm, while the maximum position error is 481 mm. In case where there is almost no slip, the distance moved by the wheel encoder is relatively accurate, but the angle measured by the gyroscope changes gradually as rotation increases. However, when a carpet (Figure 12a) is laid, the maximum error in position resulting from wheel slip is 10,346 mm, as shown in Figure 12b. In Figure 12c, when AOFS in the low-illuminance carpet environment is used, the average position error and standard deviation are 255 and 198 mm, respectively, while the maximum error is improved by 12 times from 10,346 to 873 mm without using AOFS. Analysis of the position error components shows that the error is caused by the error accumulation of the gyroscope rather than the error estimation of the moving distance by AOFS.

**Figure 13:**
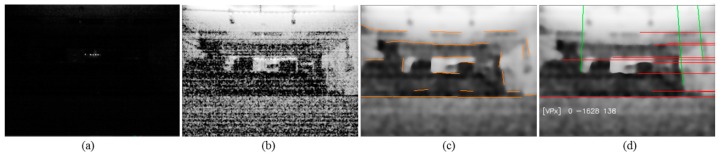
Forward viewing image of robot and image processing results obtained in 0.1 lx illumination indoor experimental environment. (**a**) Forward viewing image; (**b**) Image of histogram equalization applied to Figure 13a; (**c**) Line extracted image after rolling guidance filter applied to Figure 13b; (**d**) Vanishing point extracted from Figure 13c.

Histogram equalization is applied to the forward low-illuminance image taken every 5 cm when the robot moves or rotates 30° when a rolling guidance filter is applied. It is confirmed that the angle correction is performed by straight line extraction and vanishing point estimation, as shown in Figure 13. The maximum azimuth error is less than 1°, and the maximum position error is 0.8 m as shown in Figure 12d.

**Figure 14:**
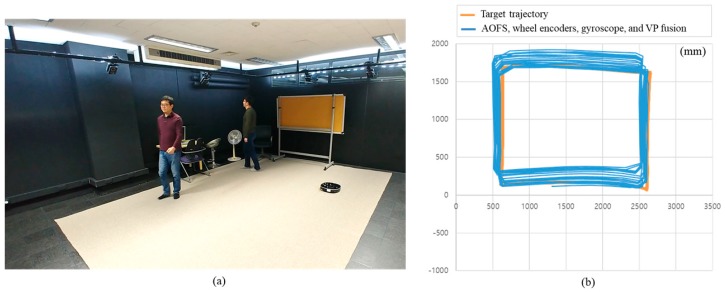
Cluttered and dynamic environments and estimated robot trajectory. (**a**) The photograph of the experimental environment; (**b**) Experimental results using the proposed method.

**Figure 15:**
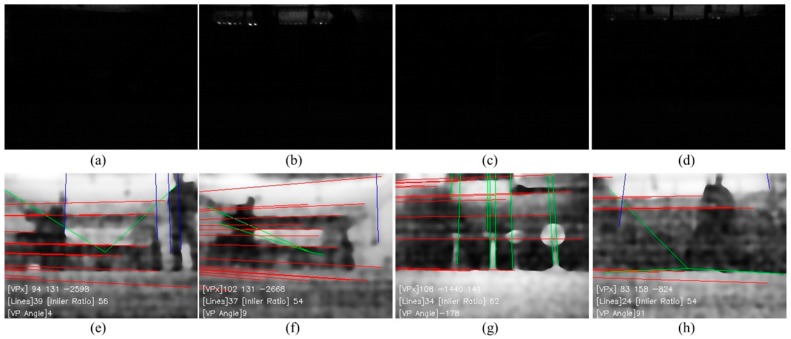
Original images and extracted VPs with the proposed method in a cluttered and dynamic environment. (**a**–**d**) The photograph of the original images taken from the robot; (**e**–**h**) The line extracted images using (**a**–**d**) respectively.

To evaluate the proposed system in a cluttered and dynamic environment, further experiments are conducted. Contrary to the previous experimental environment, several objects are placed. Also, two persons moved in the environment during the experiment. The photo of the experimental environment is illustrated in Figure 14a. Note that the lights are switched on for taking the photograph only. The actual experiment is conducted in a low-illuminance of 0.1 lx. Figure 14b shows the result of position estimation of the proposed system where the robot was driven 20 times along a 1.5 × 2.0 m rectangular path. The maximum position error is 0.8 m, and the azimuth error is less than 1°. As shown in Figure 15, the angle correction is performed by vanishing point estimation even if the images contain moving people or cluttered objects. The first row images of Figure 15 show the original images, and the second row images show extracted VPs with the proposed method.

## 5. Discussion

Conventional technologies using wheel encoders, a gyroscope, and a mono camera have less error under normal illumination, but the error increases in low illumination and slippery environments. The azimuthal correction can be done well in extreme environments by the proposed method.

It can be seen from Figure 1 that the image feature point extraction and matching performance operate at about 10 lx. However, at the 2 lx level, the error in position starts to occur because of a small number of matching feature points. Around 0.3 lx, the feature point extraction itself is not performed.

To verify the performance of conventional azimuthal correction method under general illumination, the odometry is calculated by fusing wheel encoders, a gyroscope, and an AOFS. To compensate the cumulative angular error of the gyroscope, the vanishing point is extracted from the forward-viewing image and used for angle correction. When the carpet is installed on the floor and the azimuthal correction is not performed, the error in angle is 25.6°, as shown in Figure 12c. But the experimental results show that the angular error does not increase, as shown in Figure 12d and Figure 13d, when the absolute angle correction method using vanishing point is applied even in the low illumination condition of 0.1 lx, even though there are few lines extracted as shown in Figure 13c. Even though the environment contains cluttered and moving people, the proposed system have estimated the robot trajectory as shown in Figure 14.

## 6. Conclusions

In this paper, we proposed a localization system utilizing afocal optical flow sensor (AOFS) based sensor fusion for an indoor service robot in low luminance and slippery environment where it is difficult for conventional vision-based visual odometry or SLAM to operate. A mono camera, an AOFS, wheel encoders, and a gyroscope are used for localization. The proposed system utilizes low brightness images from a mono camera to estimate the orientation of the robot. To accurately estimate the moving distance of a robot in a slippery environment, the proposed system adopts AOFS along with two conventional wheel encoders on a robot. To estimate the robot orientation, the proposed system uses a forward-viewing mono camera and a gyroscope. Since it is hard to conduct conventional feature extraction and matching in a very low luminance environment, the interior space structure from an image for robot orientation estimation is evaluated. The proposed system is developed to be operable on a low-cost processor and implemented on a consumer robot. The proposed system showed improved localization accuracy than that of conventional localization system in low illuminance and slippery environment. The proposed system is expected to be applied to various real indoor service robots in a real environment.
